# Anomaly Detection Methods in Autonomous Robotic Missions

**DOI:** 10.3390/s24041330

**Published:** 2024-02-19

**Authors:** Shivoh Chirayil Nandakumar, Daniel Mitchell, Mustafa Suphi Erden, David Flynn, Theodore Lim

**Affiliations:** 1 School of Engineering and Physical Sciences, Heriot Watt University, Edinburgh EH14 4AS, UK; sc2039@hw.ac.uk (S.C.N.); t.lim@hw.ac.uk (T.L.); 2 James Watt School of Engineering, University of Glasgow, Glasgow G12 8QQ, UK; d.mitchell.5@research.gla.ac.uk (D.M.); david.flynn@glasgow.ac.uk (D.F.)

**Keywords:** anomaly, autonomous robots, autonomous missions

## Abstract

Since 2015, there has been an increase in articles on anomaly detection in robotic systems, reflecting its growing importance in improving the robustness and reliability of the increasingly utilized autonomous robots. This review paper investigates the literature on the detection of anomalies in Autonomous Robotic Missions (ARMs). It reveals different perspectives on anomaly and juxtaposition to fault detection. To reach a consensus, we infer a unified understanding of anomalies that encapsulate their various characteristics observed in ARMs and propose a classification of anomalies in terms of spatial, temporal, and spatiotemporal elements based on their fundamental features. Further, the paper discusses the implications of the proposed unified understanding and classification in ARMs and provides future directions. We envisage a study surrounding the specific use of the term anomaly, and methods for their detection could contribute to and accelerate the research and development of a universal anomaly detection system for ARMs.

## 1. Introduction

Autonomous robotic missions (ARMs) have become increasingly important in various fields, including manufacturing [1], logistics [2], search and rescue operations [3], and even space exploration [4]. As the complexity of these missions grows, ensuring the reliability and safety of the robots becomes paramount [5]. One critical aspect of ensuring the safety and reliability of ARMs is the timely and accurate detection of anomalies [6], which can arise from various sources such as hardware faults [7], software faults [8], environmental change [9], or unexpected interactions with other systems [10,11,12,13].

Despite the significant advances in robotics and artificial intelligence (AI), detecting and identifying anomalies in ARMs remains challenging [14,15]. This is further exacerbated by the lack of a unified understanding of anomalies and the diverse range of techniques used for anomaly detection in the literature. Although there are numerous review articles on fault detection and diagnosis in robotic systems [16,17,18,19,20,21,22], they do not explicitly focus on anomalies in the context of autonomous robotic missions. Hence, the objectives of this paper are:

1. To provide a comprehensive review of the existing literature on anomalies in ARMs, covering various types of anomalies and detection methods.
2. To identify the fundamental features of anomalies in the current literature and classify them into spatial, temporal, and spatiotemporal elements.
3. To propose a unified understanding of anomalies that can serve as a basis for the development of more unified anomaly detection systems for ARMs.

The paper is organised as follows: Section 2 presents the methodology used for the literature search and selection; Section 3 explains the relationship and differences between faults and anomalies; Section 4 discusses the classification of anomalies in ARMs; Section 5 provides an overview of methods used for anomaly detection; Section 6 proposes a unified understanding of anomalies; and finally, Section 7 concludes the review and highlights future research directions.

## 2. Methodology

**Figure 1:**
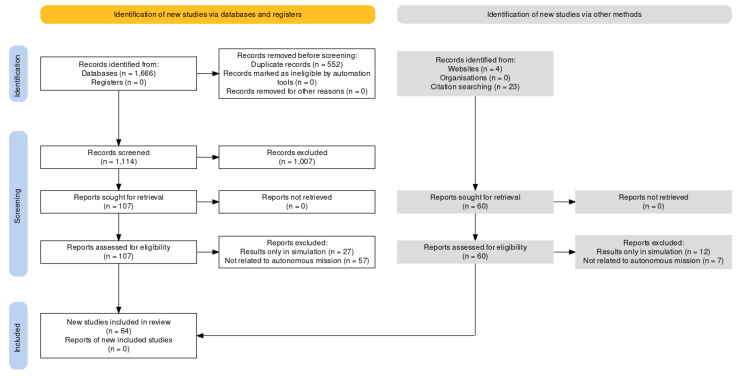
PRISMA diagram of literature search.

A systematic approach using the Preferred Reporting Items for Systematic Reviews and Meta-Analyses (PRISMA) [23] was used to guide the review. Figure 1 illustrates the workflow to establish an evidence-based minimum set of articles relevant to this review.

### 2.1. Search Strategy

We performed a systematic search of the literature using online databases, including IEEE Xplore, Scopus, and Web of Science, from inception to September 2022. The search included the following keywords and phrases: (“anomaly” OR “outlier” OR “abnormality” OR “novelty” OR “irregularity”) AND (“detection” OR “identification” OR “discovery”) AND (“autonom*” OR “robot*”). These keywords were searched within the titles, abstracts, and keywords of the papers.

### 2.2. Pre-Inclusion and Exclusion Criteria

We applied the following pre-inclusion and exclusion criteria:

Pre-inclusion criteria:

1. The paper must focus on anomaly detection or identification in the context of autonomous robotic missions (ARMs).
2. The paper must include at least one method or algorithm related to anomaly detection or identification.

Exclusion criteria:

1. Papers that discuss anomaly detection in non-autonomous systems or applications not related to robotics.
2. Papers that mention anomaly detection in ARMs only in passing, without providing any substantial discussion or contribution to the topic, such as with results only in simulation and not in a real environment.

### 2.3. Screening and Selection Process

After the initial search, the papers were screened based on their titles and abstracts to ensure they met the pre-inclusion criteria. The full text of the selected papers was then assessed for eligibility based on the exclusion criteria.

### 2.4. Data Extraction and Analysis

**Figure 2:**
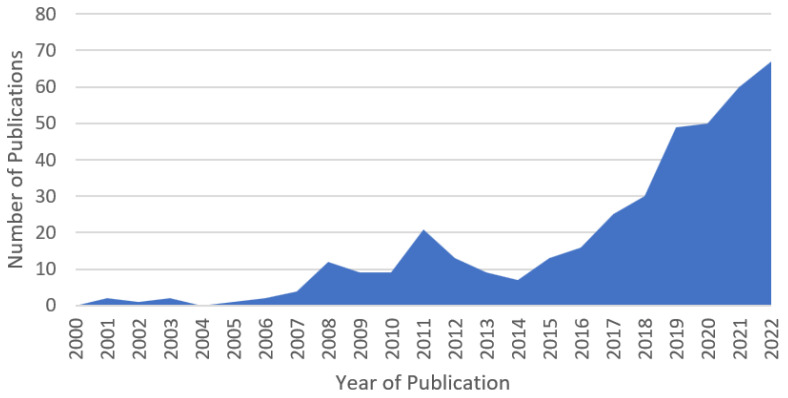
This graph was generated by counting the number of papers that matched the pre-inclusion criteria for each year. The graph displays the increasing trend of publications on anomaly detection in robotics over the years.

For each selected paper, we extracted the information on the anomaly type (spatial, temporal, or spatiotemporal), detection method, and the application relevance to ARMs. The information was then analysed and categorised to provide an overview of the current state of anomaly detection research in ARMs and to identify trends and gaps in the literature. Figure 2 indicates a 415.4% growth in publications on anomaly detection in robotics from 2015 to 2022 based on the number of papers that matched the pre-inclusion criteria.

## 3. What Are Anomalies and How Do They Differ from Faults?

**Figure 3:**
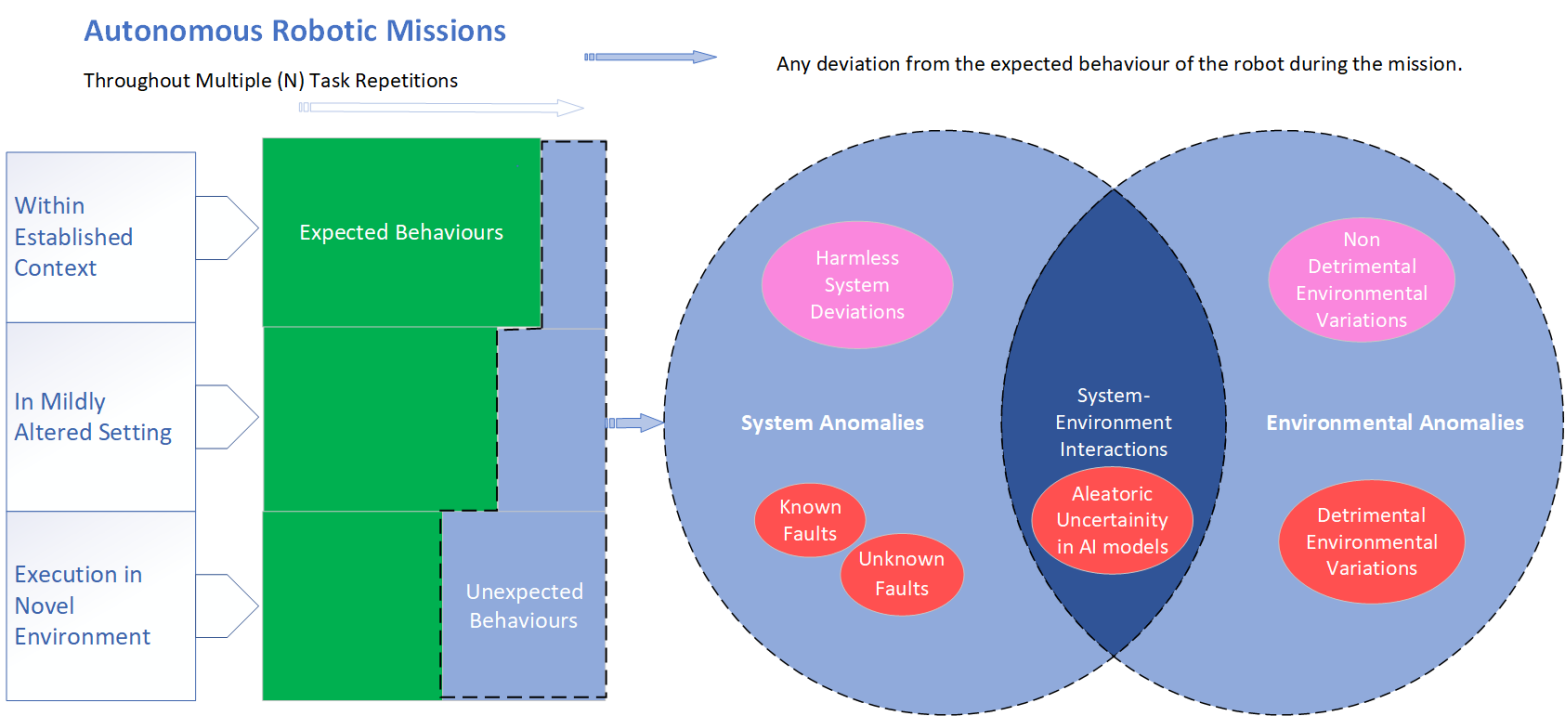
The relationship of anomalies and faults in the context of autonomous robotic missions.

The relationship between anomalies and faults is quite complex, with different researchers having varying opinions [22,24]. However, Figure 3 clarifies the fault and anomaly concepts and their relationship in the context of ARMs. When the ARM is repeated N times, the green left section of the bar represents the number of times where the hypothetical autonomous robot completes the mission with the expected behaviours. The blue bar section towards the right, represents the number of times in the mission where unexpected behaviour in execution were observed. To differentiate these unexpected behaviours, and their inter-relations, we visualise it with a Venn diagram. As observed in the Venn diagram, not all anomalies are as harmful as most people perceive [25]. If an observed anomaly was diagnosed to be harmful or disruptive to the mission continuity, it could be either due to a system anomaly or an environmental anomaly. System anomalies can be caused by faults, which can be classified as either known or unknown faults. Known faults represent the system errors, the cause of which are known and mostly have a known solution while unknown or unanticipated faults are system errors that were not experienced nor solved yet [22,24,26].

Khalastchi and Kalech’s [22] article suggests the commonly used term “unknown fault” in fault detection is indicative that there is no clear term to define the unknown faults. Such unknown faults are usually interpreted as “anomaly” in the context of ARMs. However, Graabæk et al. [27] suggests that anomalies can be symptoms of faults, implying that both known and unknown faults can be a cause of an anomaly. Moreover, we propose that the term anomaly has a meaning beyond the undesired system behaviour caused by faults and that also encapsulates the environmental impacts independent of the system and hence cannot be named as faults. Furthermore, as indicated in Figure 3, anomalies in the autonomous missions can also result from the aleatoric uncertainty of the AI models used for the perception and control tasks [28], which become predominant in interactions with specific and usually unforeseen environmental conditions to manifest unexpected behaviour, hence an anomaly.

The two coloured bars in Figure 3 highlight that the probability of the robot executing the same behaviours depends on its environment. Usually, the training of AI systems happens in a specific controlled environment. Thus, the missions will be highly repeatable in those environments; however, in new environments, there is a higher possibility that the robot will perform unexpected or wrong executions due to the probabilistic nature of certain AI models such as deep reinforcement learning and Bayesian Models [29,30,31]. Moreover, the irreducible error or aleatoric uncertainty [32], intrinsic to any AI model, is one of the reasons for anomalies in autonomous robots and is exacerbated when the robot is in a new environment [28].

The consequences of these anomalies are not always harmful to a mission, as illustrated in Figure 3. An anomaly can sometimes be inconsequential to the mission continuity. For example, a robot that uses deep reinforcement learning for motion planning can move from a start position to an end goal via different trajectories. In the presence of an environmental anomaly, and due to its interaction with the probabilistic nature of AI models, it may randomly plan a totally new path which was not experienced before; yet, the new trajectory may result in achieving the goal more or less in the same duration, hence overall it might not result in any failure or underperformance. Such deviations are considered a harmless or inconsequential anomaly.

In summary, there are different types of anomalies and different reasons for them in autonomous missions. How do we know what an anomalous behaviour is? Are there any fundamental features that define anomalous behaviours in ARMs? This is explored in the next section in reference to the reviewed literature.

## 4. Classification of Anomalies in ARMs

**Figure 4:**
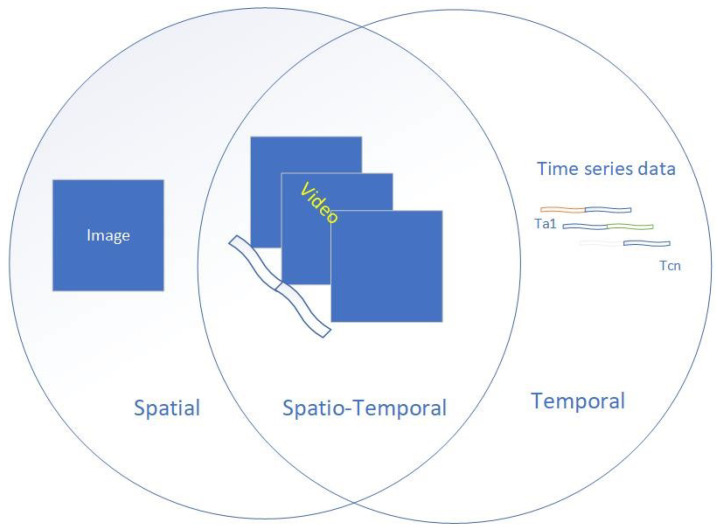
Spatial, temporal, and their correlations. For example, an image of a car with its background is purely spatial, and similarly, the time-series data showing variation in the car’s horsepower are purely temporal. However, we demonstrate the spatiotemporal correlation when the images are stacked to create a sequence of motions or when horsepower data links with the appropriate image in the sequence.

Anomalies in ARMs can manifest in various forms, with different levels of complexity and implications for the mission’s success and safety. To better understand and address these anomalies, it is essential to classify them according to their fundamental characteristics. In analysing the strategy, methods, and models within the reviewed articles, three distinct frameworks for anomalies in ARMs were identified: spatial, temporal, and spatiotemporal, as shown in Figure 4.

The spatial, temporal, and spatiotemporal classification refers primarily to the nature of the observation that defines the anomaly and its classification and, hence, the data used for its detection. For example, spatial anomalies may include hardware faults affecting the physical structure of a robot or environmental obstacles that hinder a robot’s movement. Temporal anomalies, on the other hand, may include unexpected changes in a robot’s speed, acceleration, or energy consumed over time. Spatiotemporal anomalies, which involve both spatial and temporal aspects, can be more complex and challenging to detect, as they may involve interactions between multiple robots or between a robot and its environment over time.

The distinction between these types of anomalies has implications for the methods used to detect them and the challenges faced in ARMs. For instance, spatial anomalies may be more effectively detected using image-based or distance-based algorithms, while temporal anomalies may require time-series analysis or signal-processing techniques. Spatiotemporal anomalies, due to their complexity, may require more advanced methods, such as machine learning algorithms that can capture the intricate relationships between spatial and temporal features.

Table 1 presents a summary of the key characteristics of each category of anomalies, along with representative examples of anomalies from the literature. It lists articles selected based on their relevance to the specific anomaly types, their methodological rigour, and their contributions to the understanding and detection of anomalies in ARMs.

### 4.1. Spatial Features of an Anomaly

Spatial features refer to the relationships between objects in the environment without considering the time of observation. Examples of spatial features include simple coordinates, points, lines, 3D objects, topological coverage, and linear networks [47]. In the context of ARMs, the focus is on the spatial features of objects in the environment, such as the shape of the buildings, ground, walls, and other robots.

Detecting spatial features is commonly used in the vision systems of robots, as they capture a single observation at a specific moment. For instance, Dang et al. [34] and Tomoya et al. [33] concentrate on spatial features when discussing anomalies without considering the timing of the observed behaviour. Dang et al. used drones for surveillance where objects deviating from the normal soil and bushes, such as cars or blankets, were deemed anomalous. To detect these anomalies, Tomoya et al. compared two images or static representations of the environment and identified anomalies based on variations between the observed images. Zaheer et al. [36] also focused on spatial features in their study of anomaly detection systems using moving surveillance robots with human collaboration. They employed a neural network called SiamNet to compare pairs of images, with substantial variations between the images indicating an anomaly. Although the comparison occurs at a specific point in time, the SiamNet technique does not consider the time-varying nature of the analysed data.

Resende et al. [48] offer another example: any surface variation within a pipe transporting tailings from a plant to a dam was considered an anomaly. The anomalies were detected using a deep learning model that analysed camera images of the pipe collected by the robot. In this case, the anomaly detection method and the anomalies themselves were defined solely in terms of spatial features without accounting for the timing of observations.

Lawson et al. [49] utilised generative adversarial network (GAN) models to detect spatial patterns in the environment. They identified anomalies as variations in the spatial patterns learned by the GAN models. As their focus was on identifying changes in visual observations, they did not integrate temporal relations, and their definition of anomaly was based exclusively on spatial features.

### 4.2. Temporal Features of an Anomaly

Temporal features pertain to the element of time and the patterns that emerge in a sequence of time-related observations. In ARMs, temporal features are particularly relevant due to their dynamic nature, beginning and end points, and time-bounded manipulation of objects in space. Temporal features of a robotic mission focus on patterns observed in time series data without considering the spatial relationships within the environment.

Schnell et al. [39] investigated robot anomalies by examining variations in time series data from different sensors using a Gaussian mixture model (GMM) framework. They analysed data from a robot joint when a payload was added, comparing the normal time-series pattern of the motor’s current values with the pattern during the load. They defined patterns as anomalous if they were dissimilar. Similar studies, such as anomalies in spacecraft [38], sensor anomalies in industrial robots [40], and predicting outages in collaborative robots [41], also analysed time series data and defined specific variations in the data as anomalous.

In the case of spacecraft anomalies, researchers analysed various telemetry data for five days using Long Short Term Memory (LSTM) models and dynamic thresholding to detect deviations in the usual patterns within specific time series [38]. For sensor anomalies in industrial robots, generative models were used to artificially create anomalies in the sensor data due to the scarcity of real anomaly data [40]. These pseudo-anomalies were then used to train an LSTM model for detecting variations from the normal time series.

For predicting outages in collaborative robots, the focus included how increased load could potentially lead to system failures [41]. In this case, the anomaly was the increased load, and the detection was based on the temporal variation of different parameters such as current, voltage, and temperature during the loading operation. Although the underlying cause of the anomaly was spatial (i.e., the increase in load), the detection relied on the temporal variation of parameters, leading to its classification as a temporal anomaly.

### 4.3. Spatiotemporal Features of an Anomaly

Spatiotemporal features describe the space–time relationships of events or processes, such as those occurring in ARMs. These features consider both spatial and temporal aspects of data related to objects with varying features in time within a dynamic environment.

Recent research in robotics has explored spatiotemporal correlations in various contexts. Fang et al. [45] used a graph neural network to capture spatiotemporal correlations in a scene to predict possible accidents during self-driving car missions. Another study on assistive robots in healthcare [43] initially detected spatial features such as objects in the environment. (e.g., a human as an object), Then, we further examined variations in the volume of a human object (e.g., chest movement of a human object that leads to an increase or decrease of volume), incorporating the temporal element. This research focused on spatiotemporal features and variations in these patterns to detect anomalies, such as identifying a living human in the environment.

In their robot-assisted feeding application, Park et al. [42] employed LSTM-based autoencoders to detect predefined spatiotemporal patterns. The model identified and classified anomalies by observing an increase in the reconstruction error in the current data, signalling abnormal behaviours of humans or the robot during assistive feeding experiments, such as aggressive eating or face occlusion.

Ji et al.’s [46] research on anomaly detection for robot navigation provided another perspective on spatiotemporal correlations, aiming to identify and prevent anomalies in densely cluttered outdoor robotic missions. The study first considered the usual spatial correlations in captured images, then predicted the probability of future failure by considering the planned motions from the predictive controller and the present observations from the perception module, thus incorporating the temporal element of anomalies.

In another recent paper [44], the authors claimed that their ConvLSTM models identified unusual variations in spatiotemporal elements during the motions of an autonomous vehicle and termed these variations anomalies.

In conclusion, this section has presented a classification of anomalies in ARMs by categorising them into spatial, temporal, and spatiotemporal elements, providing an understanding of the diverse nature of anomalies that may occur in autonomous robotic missions. The following section will delve into the techniques and methods employed for the detection of these anomalies in the context of ARMs.

## 5. Methods of Anomaly Detection in ARMs

This section provides an overview of the methods and techniques employed for anomaly detection in ARMs, considering the diverse types of anomalies outlined in the previous section. The discussion on anomaly detection is centred around model-based techniques and data-driven methods, emphasising the latter due to its increased adoption and scalability.

### 5.1. Model-Based Techniques

To detect anomalies, model-based techniques rely on a priori knowledge of the system’s underlying kinematics, dynamics, or other physical properties. These methods can be useful when the system’s behaviour can be accurately modelled using theoretical principles and the amount of available data is limited.

#### 5.1.1. Kinematic and Dynamic Modeling

Anomalies in the kinematic or dynamic behaviour of a robot can be detected by comparing the observed motion with the expected motion based on the system’s kinematic or dynamic models [50]. Deviations from the expected behaviour can indicate the presence of an anomaly [51].

#### 5.1.2. Model Predictive Control

Model Predictive Control (MPC) is a control strategy that uses a system model to predict future behaviour and determine the optimal control inputs. Anomalies can be identified and addressed in real-time by comparing the predicted behaviour with the actual system response [52]. However, this method needs an accurate system model, which is hard to formulate, especially when considering ARMs. Methods such as learning local linear models online have been introduced to reduce these limitations of MPC [53].

#### 5.1.3. Observer-Based Methods

Observer-based methods, such as Kalman or particle filters, have been employed to estimate a system’s internal state based on the available measurements and a system model. By comparing the estimated state with the actual state, anomalies can be detected and mitigated [54]. Similarly, hidden Markov models (HMMs) are widely used in the anomaly detection of autonomous robotic missions. Here, HMMs are used to model the normal state of the robot or different phases of the task. Once the HMM is trained, it can be used to analyze new sequences of sensor data. A low likelihood score indicates that the observed sequence is unlikely under normal operating conditions and is considered an anomaly [55]. Further, employing sliding mode observers for meticulous fault identification within robotic 271 vision systems underscores the indispensable utility of precise, model-informed diagnostics 272 in safeguarding autonomous robotic missions’ fidelity and operational efficacy [56].

The above methods heavily rely on the accurate model of either the system process or system behaviour, which is usually not available and tedious to develop due to the complexity of the system. For example, with a walking autonomous robot equipped with cameras, these approaches would require an extensive model of the behaviour of all the actuators of the robot in almost all possible states and all relevant visual information on the environmental conditions. Therefore, these methods have the limitation of not being scalable to be applied on more complex systems and to detect more complex phenomena [57].

Emerging hybrid techniques in ARMs, such as Adaptive Neural Tracking Control [58] and Generative-Model-Based Autonomous Intelligent Unmanned Systems [59], exemplify the cutting-edge integration of model-based frameworks with adaptive, data-driven technologies. While the former leverages neural networks for enhancing anomaly detection and system performance, the latter employs generative models to boost system adaptability and intelligence in dynamic environments. These approaches underscore the hybrid paradigm’s capability to transcend traditional boundaries, offering sophisticated solutions for complex challenges in autonomous system development.

### 5.2. Data-Driven Techniques

**Figure 5:**
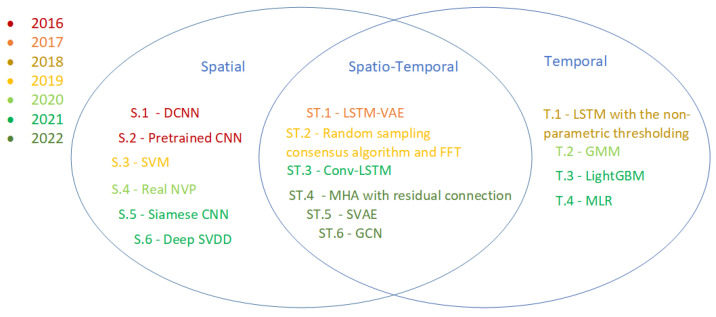
Methods to find anomalies in spatial, temporal and Spatio-temporal elements. Where S.1 [33], S.2 [34], S.3 [61], S.4 [35], S.5 [62], S.6 [26] represent spatial anomaly detection methods, ST.1 [42], ST.2 [43], ST.3 [44], ST.4 [46], ST.5 [47], ST.6 [45] represent Spatio-temporal anomaly detection methods and T.1 [38], T.2 [39], T.3 [40], T.4 [41] represent Temporal anomaly detection methods. The colour variation represents the year when the method was first used in Robotics for anomaly detection.

Data-driven techniques have been extensively used for anomaly detection in ARMs due to their minimal reliance on prior system knowledge and adaptability to data variations. In this discussion, the data-driven anomaly detection techniques are categorised according to their focus on spatial, temporal and spatiotemporal features. The review has indicated an increased utilisation of data-driven methods to detect anomalies in the recent decade. Figure 5 provides examples of various algorithms presently applied to detect spatial, temporal, and spatiotemporal anomalies in autonomous missions and with the year of the initial use of these algorithms.

The breakthrough in spatial anomaly detection happened with the progress and adoption of convolutional neural networks (CNNs) [60] and variations of the same for object recognition in the images. Spatial anomaly detection research mainly focuses on comparing the normal image with the new image and detecting any new pattern or object in the image compared to the previous one. Various algorithms, such as deep convolutional neural networks (DCNNs) [61], a convolutional neural network with multiple hidden layers, LibSVM, a library for support vector machine algorithms, and autoencoders which encode the data or, in this case, image into a latent space and detect variations in new images using the reconstruction error, is used for spatial anomaly detection. More recently, SiamNet [62] and Deep Support Vector Data Description (SVDD) [26] have been implemented where SiamNet contains two symmetric convolutional neural networks which enable the comparison of different images with minimal training data, and the Deep SVDD optimises the training data or image into a hypersphere and considers out of boundary data from this distribution as anomalies.

The main challenge in temporal anomaly detection is developing models or systems that can remember past experiences or quantify patterns from long and complex time-series data. The temporal anomaly detection problem is not equivalent to sensor noise prediction problems. The noise, or any sudden amplitude or frequency change in the signal, can be easily captured or filtered out using already established digital signal processing methods such as Fast Fourier Transform (FFT) [63] and Radon Fourier Transform (RFT) [64], or cancelled out via the feedback controllers inside the autonomous robots. The main breakthrough leading to temporal anomaly detection through solving and capturing patterns in highly complex time series data in robotic systems emerged with the development of recurrent neural networks (RNNs) [65]. RNNs are neural networks capable of remembering past experiences or storing helpful information from past data, and LSTM [66] is a type of RNN widely used in time series forecasting.

Moreover, the Gaussian mixture model (GMM) [67] is used for health monitoring and detecting anomalies from the sensors in the robots, wherein the healthy robot data is used for training the model that is then able to detect any anomalies in the time series data without any previous knowledge of the anomalies. Light Gradient Boosting Machine or LightGBM, a fast and high-performing gradient boosting algorithm, has recently been used for time series data analysis [39]. It is similar to the multiple linear regression (MLR) method used in time series forecasting in robotics systems [40].

Spatio-temporal anomaly detection targets anomalies with spatial and temporal correlations and hence is more challenging due to the complexity of finding the spatial correlations, temporal correlations and the correlations between both. Recent advancements in spatio-temporal anomaly detection have stemmed from the integration of convolutional networks in the RNNs to create Convolutional LSTMs (Conv-LSTM) [11] and Integrating Variational Autoencoders with LSTMs. Both these frameworks were able to capture the spatial variations, temporal variations, and correlations. For example, capturing anomalies by collecting various sensor data such as from the Global Navigation Satellite System (spatial), the location, position, and orientation of an autonomous car in a specific time interval (temporal) is a representative of spatio-temporal anomaly detection [44]. In Figure 5, ST.2 mentions using FFT in anomaly detection. Here, spatial anomaly (human in a specific location) is captured via a Random Sampling consensus algorithm, and further, FFT is specifically used to detect the variation in the chest movement of the detected human. Thus, the overall system can detect spatiotemporal anomalies. J. W. Kaeli et al. focuses on a data-driven approach that uniquely combines spatial and temporal anomaly detection aspects through semantic mapping in the context of autonomous underwater vehicles [68]. Given its emphasis on real-time processing of diverse data types, such as optical and acoustical imagery for dynamic environmental interpretation and anomaly identification, it naturally extends into spatiotemporal anomaly detection.

In summary, this section has provided an overview of the model-based and data-driven techniques employed for anomaly detection in ARMs. We have specifically focused on the data driven detection methods targeting spatial, temporal, and spatio-temporal anomaly detection, which are mainly based on application of deep neural networks.

## 6. Towards a Unified Understanding of Anomaly in ARMs

In light of the presented literature review, we posit and introduce inferential constituents that define anomalies in the context of ARMs. The discussion henceforth is to establish a unified understanding.

### 6.1. *What Is Anomaly in ARMs?*

An anomaly in ARMs is a deviation from the:

- Expected behaviour and performance of a robotic system;
- Expected sequence of states of the robotic system during its operation;
- Expected form and mode of interaction with its environment;

Which may impact the mission’s objectives, safety, or efficiency. The expected behaviour, performance, or state transitions are defined by the robot’s design, control algorithms, and mission constraints.

### 6.2. In What Form Can Anomaly Be Observed?

Anomalies can be observed in spatial, temporal, or spatio-temporal forms. This form depends on the nature of the data or the patterns through which the autonomous robot is monitored and through which the detection is implemented.

### 6.3. What Is the Source of Anomaly?

Anomalies can arise from the robotic system itself (e.g., sensor errors, actuator malfunctions) or from the environment (e.g., unexpected obstacles, changing conditions). The source of an anomaly can be a system failure as well as an unforeseen mode of interaction with environment.

### 6.4. How Severe Are Anomalies?

Anomalies can range from minor deviations that do not significantly impact the mission’s objectives to critical faults that may lead to system failures or safety hazards.

With these descriptions, we aim at progressing towards a unified understanding of the concept of anomaly in ARMs, and delineating the detection frameworks that apply to different categories of anomalies. Such unified understanding might facilitate the comparison and integration of various anomaly detection techniques and promote the advancement of safe and reliable autonomous robotic systems.

## 7. Conclusions

This review comprehensively examined the literature on anomalies in Autonomous Robotic Missions (ARMs). Our exploration of the literature highlighted the diversity in approaches and definitions of anomalies within autonomous robotics.

We introduced a classification of anomalies into spatial, temporal, and spatiotemporal categories, focusing on the domains in which anomalies manifest. This classification has been closely linked to the methods employed to detect anomalies in literature. We provided a discussion of methods of anomaly detection and highlighted the prominent ones that apply to the detection of spatial, temporal, and spatiotemporal anomalies: image processing-based algorithms more generally apply to spatial anomalies, sequential data analysis and signal processing techniques are more relevant for temporal anomaly detection, and more advanced machine learning techniques are applied to simultaneously capture the correlations of spatial and temporal features in spatiotemporal anomalies. We reviewed both data-driven and model-based techniques to achieve these goals.

Moreover, we formulated a set of descriptions of anomalies in ARMs, which can be gathered into a working definition as follows: An anomaly in Autonomous Robotic Missions is a deviation from the expected behaviour, performance, or state of the robotic system and its environment, which may impact the mission’s objectives, safety, or efficiency; and this anomaly can be caused either by system faults or the change in the environmental dynamics of interaction. The nuanced understanding of anomaly categories facilitates a more strategic approach, ensuring that detection methods are more effective in addressing the specific nature of the anomaly.

This review shows that integrating data-driven and model-based techniques is an emerging avenue for future research, as challenges in anomaly detection cannot be solved solely using data or predefined system models. The potential of this integration to enhance anomaly detection in ARMs underscores the need for concerted efforts to overcome the inherent challenges of combining heterogeneous sources of information and analytical techniques.

Furthermore, this review highlights that ARMs involve physical systems operating in dynamic real-world environments, necessitating predictability and understandability to foster trust among operators and stakeholders. The ability to provide clear insights into the system’s decision-making processes, especially in detecting and managing anomalies, is crucial for ensuring these missions’ safety, efficiency, and reliability. This requirement for transparency extends beyond technical necessity, becoming a cornerstone for the ethical deployment and societal acceptance of autonomous systems. The implications of the review span various sectors, including automotive, service industries, self-driving vehicles, autonomous drones, and underwater robotics. Benefiting from the review insights, stakeholders across these sectors can enhance the safety and reliability of their autonomous systems by adopting the best practices, designing new detection methodologies and ensuring ethical deployments, ultimately driving the evolution of autonomous technologies in real-world applications.

## Figures and Tables

**Table 1:** Features of anomalies observed in ARMs.

Title	Year	“Anomaly” Determinants	Data Used to Capture “Anomalies”	Anomaly Class
Compressive change retrieval for moving object detection [33]	2016	The difference in the given image and the previous similar images retrieved from a search.	GPS data, two cameras, and LiDAR data	Spatial
Anomaly detection and cognisant path planning for surveillance operations using aerial robots [34]	2019	Car, blanket	Camera of a DJI Matrice 100	Spatial
Safe robot navigation [35]	2020	Variation in environmental conditions (Sunlight, Fire, Rain, Wet)	RGB-D, Gravity Aligned Depth. Gravity Aligned Surface Normals. Gravity Aligned Surface Normals.	Spatial
An anomaly detection system via moving surveillance robots with human collaboration [36]	2021	Variation of the position of objects in the reference images to that of the observed image.	RGB data from a camera attached to the robot	Spatial
An anomaly detection approach to monitor the structure-based navigation in agricultural robotics [37]	2021	Low light, shadows, leaf-covering sensors, and unrealistic basic assumptions in the tracking algorithms	16-channel 3D LiDAR sensor	Spatial
Curiosity MSL [38]	2018	Variation in the individual telemetry data.	Telemetry data for a specific time frame (5 days) nearby an anomaly	Temporal
Robot health estimation through unsupervised anomaly detection using gaussian mixture models [39]	2020	Robot immobilised or unstable due to external influences such as extra payload	Current from the motor	Temporal
Anomaly detection in industrial robots [40]	2021	Overload, parts breaking, environmental effects, maloperation, program exceptions, transmission errors	Power factor. Loop current. Reactive power. Active power. Current, Voltage. Incoming frequency.	Temporal
Anomaly detection in cobots [41]	2021	Increase in temperature due to load and speed of the cobot during human-robot interaction.	Joint values, Speed, Current, Voltage Power	Temporal
Robot-assisted feeding [42]	2017	Touch by a user, aggressive eating, utensil collision by a user, sound from a user, face occlusion, utensil miss by a user	RGB-D, Joint torque, Sound energy	Spatio-Temporal
Human-care rounds robot with contactless breathing measurement [43]	2019	A breathing Human on the floor	RGB-D and Thermal Point Cloud (TPC)	Spatio-temporal
Anomaly detection using IoT sensor-assisted ConvLSTM models for connected vehicles [44]	2021	Unusual Variations in autonomous vehicle navigation and powertrain data	Temperature sensors, pressure sensors, location and orientation sensors	Spatio-temporal
Traffic accident detection via self-supervised consistency learning in driving scenarios [45]	2022	Inconsistent movements of humans and other vehicles	RGB	Spatio-temporal
Proactive anomaly detection for robot navigation with multi-sensor fusion [46]	2022	Weeds and low-hanging leaves block the sensory signals.	RGB, Lidar point cloud, and the planned path.	Spatio-temporal
